# Evaluation of a new calibration index suggesting recalibration of the pulse contour cardiac index by transpulmonary thermodilution: a prospective study

**DOI:** 10.1186/cc12126

**Published:** 2013-03-19

**Authors:** W Huber, K Waldleitner, S Mair, B Saugel, RM Schmid

**Affiliations:** 1 Klinikum rechts der Isar, Technical University of Munich, Germany

## Introduction

After calibration by thermodilution (TD), the PiCCO device is able to assess cardiac index (CI) using pulse contour (PC) analysis. The manufacturer suggests recalibration by TD after 8 hours. Recently, we suggested a calibration index indicating a certain probability of a relevant bias and triggering the next calibration. With changes of CIpc compared with the previous CItd being a key predictor of the bias of CIpc compared with the following CItd, the manufacturer implemented a soft alarm indicating changes in CIpc compared with the last CItd (thresholds adjustable to 15%, 25% and 35%). The aim of the study was prospective evaluation of predictive capabilities of the 15% alarm regarding a bias of CIpc compared with CItd exceeding critical thresholds of 15%, 20% and 25%.

## Methods

A prospective analysis of 329 routine TPTD measurements in 70 patients. The CI-trend alarm was set to 15%, and CIpc, trend alarm (yes/no), relative and absolute changes in CIpc were recorded immediately before TD providing exact measurement of CItd. Predictive capabilities of the 15% trend alarm regarding the bias were evaluated using Spearman correlation, chi-square test, ROC analysis and Wilcoxon test (IBM SPSS 20).

## Results

A total of 70 patients (24 female, 46 male), age 62 ± 14 years, APACHE II score 19.5 ± 7.5, SOFA score 8.9 ± 4.4. At 937 ± 904 (50 to 5,795) minutes after the last CItd, CIpc provided a bias of -0.0566 ± 0.761 l/ minute*m^2 ^compared with the next CItd. Percentage error was 34.6%. Absolute and relative bias of CIpc compared with the new CItd did not correlate to time to last TD, but correlated (all *P <*0.001) to CIpc itself (*r *= 0.333; *r *= 0.385) and relative (*r *= 0.531; *r *= 0.537) and absolute (*r *= 0.533; *r *= 0.529) changes in CIpc compared with last CItd. The 15% CIpc trend alarm was indicated before 83/329 measurements (25.2%). The amount of bias exceeded 15% and 20% in 101 (30.7%) and 79 (24%) of TDs. In TDs with indicated trend alarm (≥15% deviation of CIpc to the last CItd), the amount of bias more frequently exceeded 15% (*P *= 0.019), 20% (*P <*0.001) and 25% (*P <*0.001). Time to last calibration ≥8 hours was not associated with bias exceeding 15% (*P *= 0.735), 20% (*P *= 0.888) or 25% (*P *= 281).

## Conclusion

Bias of CIpc compared with the next CItd does not depend on the time since last CItd. Changes in CIpc itself compared with the last CItd are associated with the bias. A 15% change in CIpc trend alarm provided by the new PiCCO algorithm is significantly associated with bias values exceeding 15%, 20% or 25% of CItd, whereas time to last calibration >8 hours is not associated with bias exceeding these critical thresholds.

